# The Mural Decoration of Hospitals

**Published:** 1912-06-29

**Authors:** 


### THE MURAL DECORATION OF HOSPITALS.

In our columns last year (September 23) fresco decoration for walls was suggested as a new departure which, while giving increased interest to plain surfaces, would at the same time ensure a hygienic and cleanly appearance to the ward, and if the subjects be well chosen should be inspiring to patients both during and after their recovery.

That such an idea has already found root may be gathered from the report of the meeting of painters in tempera on June 18, when eminent authorities tried to rouse enthusiasm in this decorative art. Though tempera decoration can be dated as far back as 4500 B.C., when it had attained a high pitch of completeness, it has for the last 200 years been in a state of comparative quiescence.

This may probably be in some manner accounted for by the ignorance of artists in technical knowledge; for it must not be forgotten that the life of fresco is affected both by preliminary details of building construction and by the methods employed in the actual painting. Damp is one of the chief causes of decay, and if the wall be not kept thoroughly dry painting upon it is sure to deteriorate; changes in colours caused by the action of lime in the plaster or by injurious gases in the atmosphere have also to be guarded against. Careless laying of the surface plaster also begets cracks and encourages peeling; it is in avoiding these defects of mechanism that the artist has first to be educated.

The careful preparation and ageing of surface plaster is one of the secrets of the longevity of the frescoes of Italy's golden days. It was not uncommon for an artist to use material made fifty or more years before his time. As the colours of work done by the Egyptians 4,000 years ago still show bright and vivid, it may safely be inferred that they also thoroughly understood their medium.

To discuss the relative merits of different plasters is beyond the province of this article; suffice it to say that not only have we better knowledge than had the ancients or the mediævalists, but we have also a more extensive chromatic scale than Angelo had when the wonderful ceiling of the Sistine Chapel was painted. There should, therefore, on this score, be lacking no enthusiasm in the present-day student.

### Some Existing Examples.

In mediæval days the Gothicists decorated most of their interiors in colour, so that the buildings did not at all resemble the grey interiors as we now know them; a good idea of what a Gothic interior was like may, however, be formed by visiting Holloway Sanatorium at Virginia Water, where the detail and colours are particularly good. Another interesting example of mural decoration is to be seen in a cubicle at Hanwell Mental Hospital, done entirely by a patient some years ago. In the ward generally, however, the fresco decoration should be confined to the upper wall surfaces, for the lower parts must, of necessity, be more frequently re-decorated for aseptic reasons. The pavilion of the future will no doubt present larger windows and smaller wall surfaces than we are at present accustomed to; thus frescoes will at times be in more or less direct sunlight and at other times they will be artificially illuminated. This is an important point which must not be forgotten.

### The Qualities of the Mural Artist.

Fresco in its true sense is limited to work upon a very thin skin of plaster which has been newly laid. The mineral colours are laid on the damp surface so that they soak into and become solidly incorporated with the base. It depends for its durability not upon thorough drying, but upon the crystallisation of the carbonate of lime on the surface into a fine transparent protecting enamel. The colours must be such that they will not suffer from contact with lime, and it can readily be understood that the work must be "kept going" so that it will not show seams.

The *chefs d'œuvre* of painting are in oils, the grandest compositions are in fresco; it requires thorough draughtsmanship, skilful composition, a thorough grasp of the relative value of line to mass, a true feeling for harmony in colour, and an exceptional grandeur of style; in short, the mural artist must possess all the qualities of a good picture-painter.

The subjects chosen must be of an inspiring nature; this quality may be felt in the King's robing-room frescoes at Westminster, taken from *Morte d'Arthur* and representing Courtesy, Religion, Generosity, Hospitality, and Mercy. The whole idea of subject may be summed up in the true words of Cicero: "The object of all art is to soften the manners by training the heart and mind to right thoughts and worthy sentiments."

[This article should be contrasted with the other aspects of the question which are dealt with in the article on the opposite page.—Editor, "The Hospital."]

